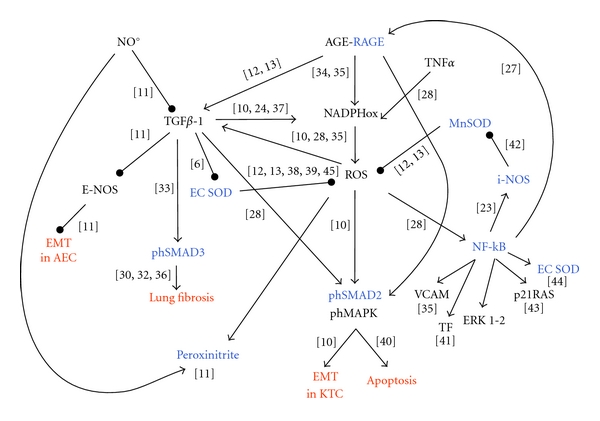# Erratum to “Factors Influencing Oxidative Imbalance in Pulmonary Fibrosis: An Immunohistochemical Study”

**DOI:** 10.1155/2011/515608

**Published:** 2011-08-01

**Authors:** S. Inghilleri, P. Morbini, I. Campo, M. Zorzetto, T. Oggionni, E. Pozzi, M. Luisetti

**Affiliations:** ^1^Respiratory Disease, IRCCS Policlinico San Matteo Foundation, University of Pavia, Pavia, Italy; ^2^Department of Pathology, IRCCS Policlinico San Matteo Foundation, University of Pavia, Pavia, Italy

The authors of the paper would like to apologize for the following errors contained in the original paper.

1. The exact Figure 1 in the original paper has to be corrected as Figure 1 in this paper. 

2. References in the original paper have to be corrected by adding the following:

- M. D. Oldfield, L. A. Bach, J. M. Forbes et al., “Advanced glycation end products cause epithelial-myofibroblast transdifferentiation via the receptor for advanced glycation end products (RAGE),” *Journal of Clinical Investigation*, vol. 108, no. 12, pp. 1853–1863, 2001.
- M. P. Wautier, O. Chappey, S. Corda, D. M. Stern, A. M. Schmidt, and J. L. Wautier, “Activation of NADPH oxidase by AGE links oxidant stress to altered gene expression via RAGE,” *American Journal of Physiology*, vol. 280, no. 5, pp. E685–E694, 2001.
- J. Zhao,W. Shi, Y. L.Wang et al., “Smad3 deficiency attenuates bleomycin-induced pulmonary fibrosis in mice,” *American Journal of Physiology*, vol. 282, no. 3, pp. L585–L593, 2002.
- V. J. Thannickal and B. L. Fanburg, “Activation of an H2O2-generating NADH oxidase in human lung fibroblasts by transforming growth factor **β**1,” *Journal of Biological Chemistry*, vol. 270, no. 51, pp. 30334–30338, 1995.
- A. Bellocq, E. Azoulay, S. Marullo et al., “Reactive oxygen and nitrogen intermediates increase transforming growth factor-**β**1 release from human epithelial alveolar cells through two different mechanisms,” *American Journal of Respiratory Cell and Molecular Biology*, vol. 21, no. 1, pp. 128–136, 1999.
- M. H. Barcellos-Hoff and T. A. Dix, “Redox-mediated activation of latent transforming growth factor-**β**1,” *Molecular Endocrinology*, vol. 10, no. 9, pp. 1077–1083, 1996.
- C. L. Fattman, “Apoptosis in pulmonary fibrosis: too much or not enough?” *Antioxidants and Redox Signaling*, vol. 10, no. 2, pp. 379–385, 2008.
- A. Bierhaus, T. Illmer, M. Kasper et al., “Advanced glycation end product (AGE)-mediated induction of tissue factor in cultured endothelial cells is dependent on RAGE,” *Circulation*, vol. 96, no. 7, pp. 2262–2271, 1997.
- V. Nilakantan, N. L. N. Halligan, T. K. Nguyen et al., “Post-translational modification of manganese superoxide dismutase in acutely rejecting cardiac transplants: role of inducible nitric oxide synthase,” *Journal of Heart and Lung Transplantation*, vol. 24, no. 10, pp. 1591–1599, 2005.
- H. M. Lander, J. M. Tauras, J. S. Ogiste, O. Hori, R. A. Moss, and A. M. Schmidt, “Activation of the receptor for advanced glycation end products triggers a p21(ras)-dependent mitogen-activated protein kinase pathway regulated by oxidant stress,” *Journal of Biological Chemistry*, vol. 272, no. 28, pp. 17810–17814, 1997.
- R. J. Folz and J. D. Crapo, “Extracellular superoxide dismutase (SOD3): tissue-specific expression, genomic characterization, and computer-assisted sequence analysis of the human EC SOD gene,” *Genomics*, vol. 22, no. 1, pp. 162–171, 1994.
- L. I. Wang, D. P. Miller, Y. Sai et al., “Manganese superoxide dismutase alanine-to-valine polymorphism at codon 16 and lung cancer risk,” *Journal of the National Cancer Institute*, vol. 93, no. 23, pp. 1818–1821, 2001.

## Figures and Tables

**Figure 1:**